# Sagittal balance dynamics during gait in patients with degenerative spine disease

**DOI:** 10.1007/s43390-026-01347-5

**Published:** 2026-04-02

**Authors:** Armand Dominik Škapin, Janez Vodičar, Matej Supej, Nina Verdel, Miha Vodičar

**Affiliations:** 1 Department of Orthopaedic Surgery, University Medical Centre Ljubljana, Zaloška Cesta 9, 1000 Ljubljana, Slovenia; 2 Faculty of Medicine, University of Ljubljana, Vrazov Trg 2, 1000 Ljubljana, Slovenia; 3 Faculty of Sport, University of Ljubljana, Gortanova Ulica 22, 1000 Ljubljana, Slovenia

**Keywords:** Degenerative spine disease, Spinal deformity, Sagittal balance, Gait analysis, Motion capture, Compensatory mechanisms

## Abstract

**Study Design:**

Observational Case–Control Study.

**Purpose:**

To evaluate the dynamics of sagittal balance during gait in patients with degenerative spine disease compared with matched controls.

**Methods:**

33 patients with pathological sagittal balance parameters on standing radiographs and 31 matched controls underwent three-dimensional motion capture analysis during a six-minute walking assessment. Data were collected across five phases: pre-walk, initial-walk, mid-walk, end-walk, and post-walk. Spino-pelvic (pelvic angle, lumbar lordosis, thoracic kyphosis) and sagittal balance parameters (sagittal trunk shift and angle, global tilt), as well as lower limb joint angles were assessed.

**Results:**

The greatest decrease in sagittal balance occurred during the transition from standing to walking, with significantly larger changes observed in the study group (C7-L5-sagittal trunk shift increasing from 69.1 ± 50.6 mm to 138.7 ± 51.9 mm) compared to controls (increase from 20.9 ± 18.8 mm to 69.2 ± 19.7 mm; p = 0.020). The decrease was primarily due to increased thoracic kyphosis and, in some cases, reduced pelvic retroversion. During continued walking, sagittal balance remained stable at the group level, with only a subgroup in both groups demonstrating a notable decrease. No significant differences in changes during walking were found between groups. After walking, parameters largely returned close to baseline in a standing position, indicating an almost complete recovery of sagittal balance.

**Conclusions:**

Compensatory mechanisms for maintaining sagittal balance are significantly more effective in a standing position than during walking. While sagittal balance remains stable in most patients during short-term walking, a notable decrease occurred in several individuals. Therefore, further research is needed to identify prognostic factors associated with a decrease in sagittal balance during walking.

## Background

The prevalence of degenerative spine disease is increasing with the aging population, often resulting in altered spinal curvatures such as loss of lumbar lordosis [1, 2]. These changes can disrupt sagittal balance, which is crucial for efficient upright posture [3–5]. Clinically, sagittal balance is assessed using full-spine standing sagittal X-rays, capturing only a brief moment of forced upright stance. However, this method may not accurately represent a patient’s true sagittal balance, as compensatory mechanisms can temporarily mask underlying imbalances [3, 4, 6–12]. On the other hand, these compensatory mechanisms can become compromised during dynamic activities due to muscle fatigue [7–12]. Thus, accurate evaluation of sagittal balance in patients with degenerative spine disease requires analysis of gait dynamics.

Several methods for dynamic assessment of sagittal balance using motion capture systems have already been proposed [7–12]. However, many had methodological limitations, such as using treadmills instead of solid ground, short walking distances, small sample sizes, or less sophisticated motion tracking technology. They also often focused on a limited set of parameters, neglecting a comprehensive evaluation of sagittal balance [7–12]. To address these limitations, we developed a comprehensive protocol for the assessment of sagittal balance during gait. This method showed moderate-to-excellent reliability in older adults with back pain [13].

With this study, we aimed to evaluate sagittal balance dynamics during gait in patients with radiographic sagittal imbalance (pathological PI-LL or SVA) and compare them with controls. Such analysis may be essential for improving clinical assessment and identifying the primary compensatory mechanisms involved.

## Participants and methods

### Participants

**Fig. 1:**
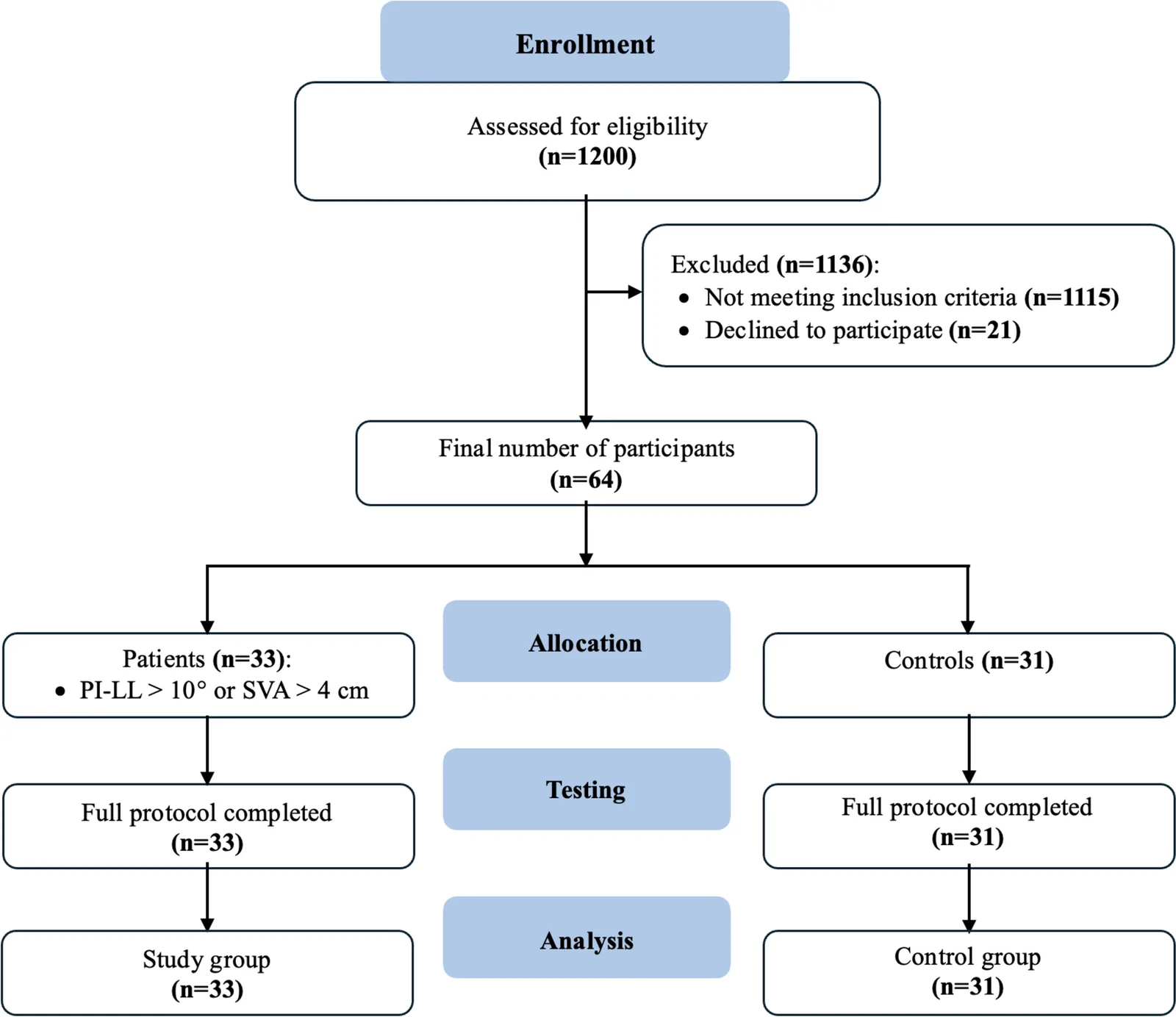
Flowchart of participant recruitment in the study

Participants were recruited from an orthopedic spine surgery outpatient clinic at a single university medical center. The enrollment process is presented in Fig. 1.

Inclusion criteria were (1) PI-LL mismatch greater than 10° or SVA greater than 4 cm, (2) back pain as the main symptom, and (3) age between 50 and 80 years. Exclusion criteria were (1) symptoms of spinal stenosis and radiculopathy, (2) previous instrumented spinal surgery, (3) a coronal Cobb angle greater than 30°, (4) symptoms of hip or knee arthrosis, (5) symptoms of vascular intermittent claudication, (6) cardio-pulmonary disease that impairs the patient’s ability to walk for six minutes, and (7) neuromuscular disease.

Following enrollment, participants underwent standing full-spine sagittal X-rays to assess spino-pelvic and sagittal balance parameters. Participants with positive SVA and/or PI-LL mismatch were assigned to the study group. A control group was formed using identical inclusion and exclusion criteria, except their SVA and PI-LL mismatch values were within the physiologic ranges (PI-LL < 10°, SVA < 4 cm) (Fig. 1).

## Motion capture system and marker placement

**Fig. 2:**
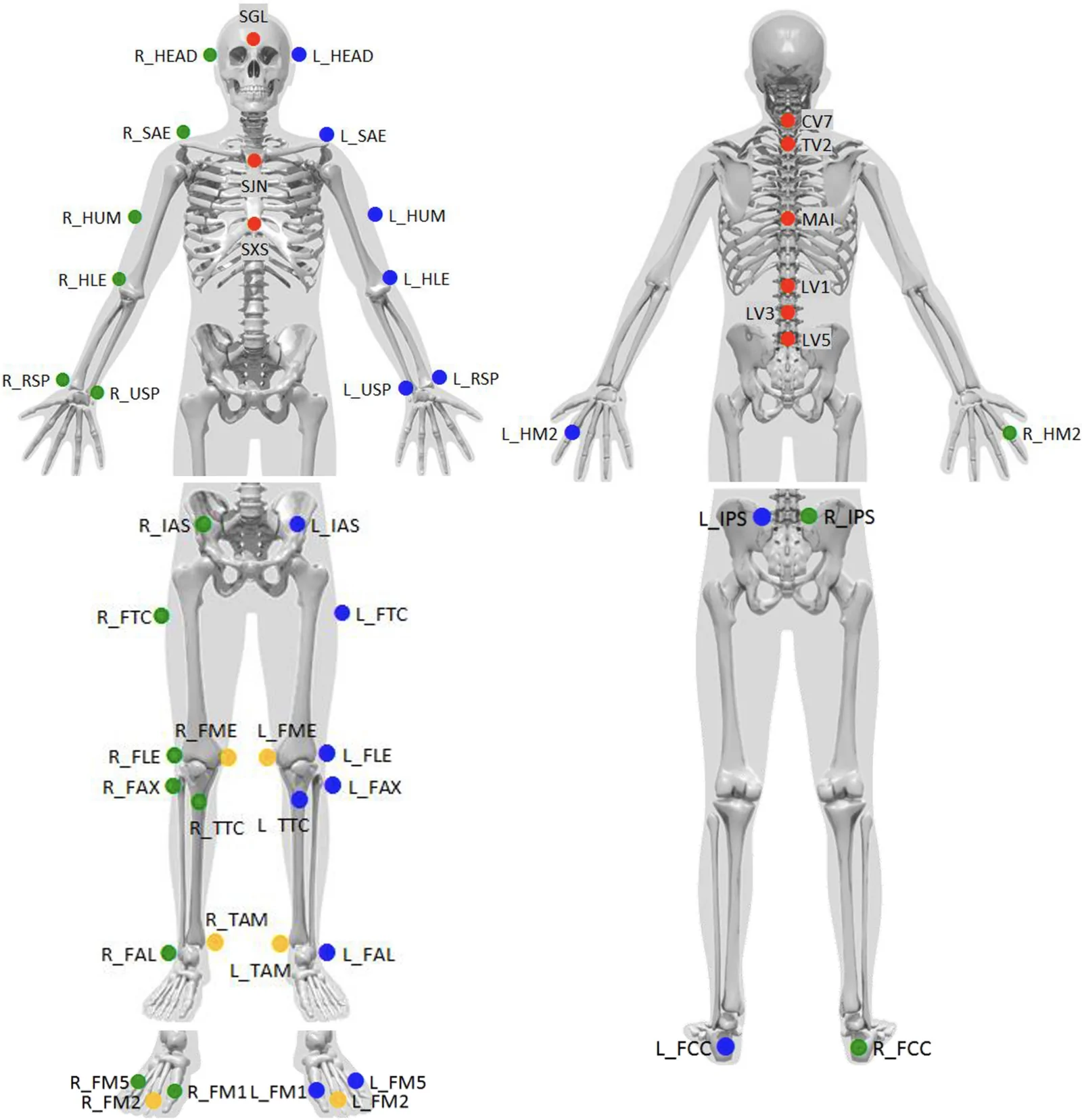
Placement positions of 49 reflective markers based on the “Qualisys PAF package: Instituti Ortopedici Rizzoli (IOR).” This standard image from Qualisys illustrates marker placement as defined in the official protocol

Gait analysis was performed using an optoelectronic Motion Capture System (Qualisys AB, Göteborg, Sweden) comprising twelve Oqus 7 + cameras strategically arranged around the testing space. 49 reflective markers were placed directly onto the skin of each participant while they were seated following the “Qualisys PAF package: Instituto Ortopedico Rizzoli (IOR)” protocol (Fig. 2) [14–16]. All markers were placed by the same researcher to ensure consistent placement, utilizing palpation to accurately identify each bony landmark.

## Walking protocol

Participants walked barefoot for six minutes between two cones positioned seven meters apart, wearing minimal clothing and walking at a normal, self-selected pace.

Gait was recorded during five phases:

- Pre-walk measurement: Standing baseline posture matching the position used for full-spine sagittal X-ray imaging. Participants held a fabric band draped around their necks with both hands, resulting in slight shoulder anteflexion and elbow flexion.
- Initial-walk: During the first 30 s of walking, three consecutive steps (each defined as movement between two heel strikes) from straight-line segments were selected for analysis, ensuring complete marker visibility.
- Mid-walk: A 30-s gait recording was taken at the midpoint, and three consecutive steps were selected for analysis.
- End-walk: The final 30 s of walking were recorded, and three consecutive steps were analyzed.
- Post-walk measurement: After completing the six-minute walking period, participants stood still again for a final static recording.

## Spino-pelvic and sagittal balance parameters

**Fig. 3:**
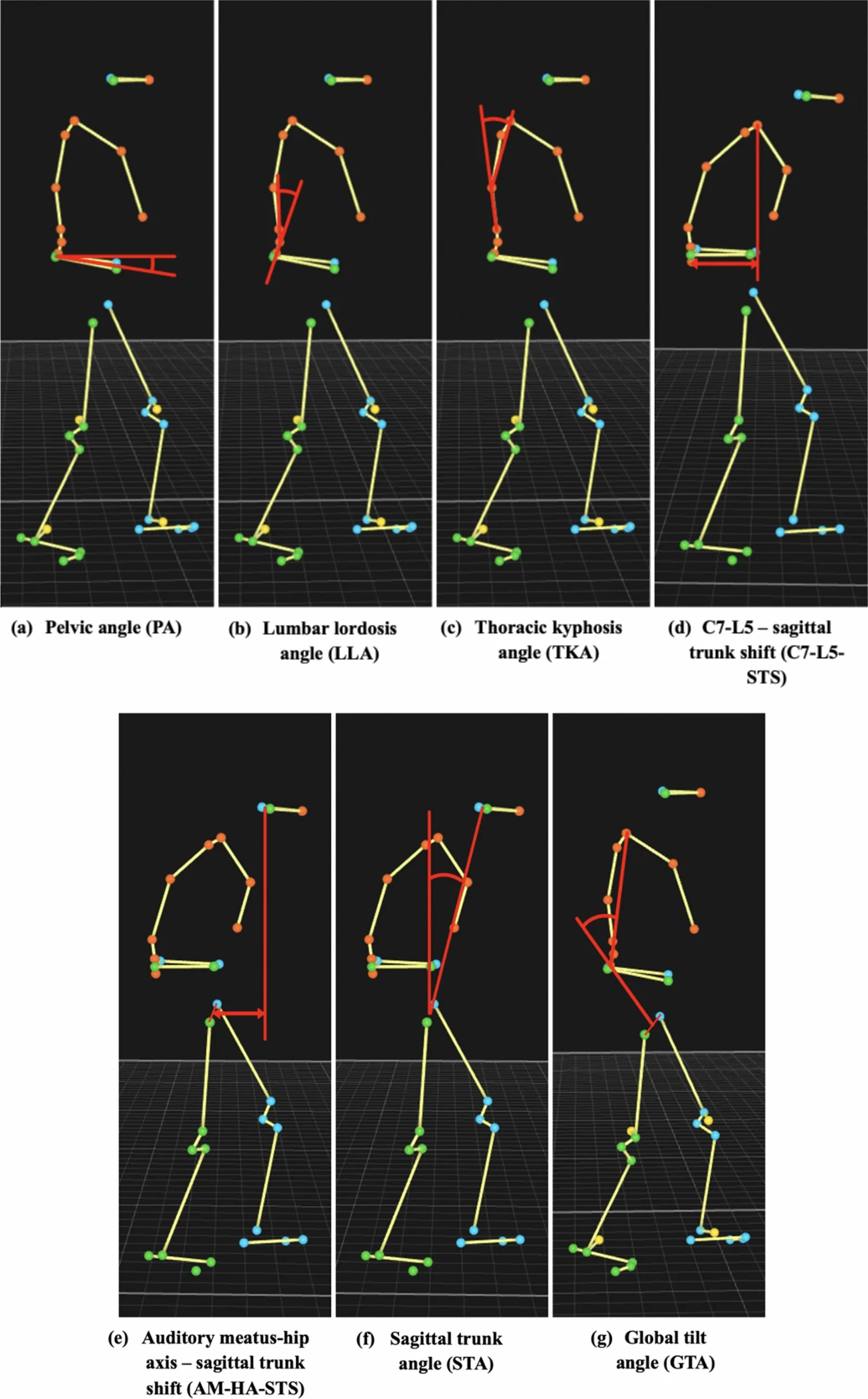
A 2D schematic representation of the measured parameters

During each of the five phases, the following parameters were measured:

- Pelvic angle (PA): Angle between the horizontal plane and the line connecting the midpoints of markers on the anterior and posterior superior iliac spines (Fig. 2: markers L_IPS, R_IPS, L_IAS, R_IAS; Fig. 3a).
- Lumbar lordosis angle (LLA): Angle between two lines connecting markers on the spinous processes of L1, L3, and L5 (Fig. 2: markers LV1, LV3, LV5; Fig. 3b).
- Thoracic kyphosis angle (TKA): Angle between two lines connecting markers on the spinous process of C7, at the inferior edge of the scapulae, and L1 (Fig. 2: markers CV7, MAI, LV1; Fig. 3c).
- C7-L5-sagittal trunk shift (C7-L5-STS): Horizontal distance between vertical lines passing through markers on the spinous process of C7 and L5 (Fig. 2: markers CV7 and LV5; Fig. 3d).
- Auditory meatus-hip axis-sagittal trunk shift (AM-HA-STS): Horizontal distance between vertical lines passing through the midpoints of markers placed superior to both ears and markers placed on the greater trochanters (Fig. 2: markers R_HEAD, L_HEAD, R_FTC, L_FTC; Fig. 3e).
- Sagittal trunk angle (STA): Angle between a vertical line and the line connecting the midpoints of markers superior to both ears and markers on the greater trochanters (Fig. 2: markers R_HEAD, L_HEAD, R_FTC, L_FTC; Fig. 3f).
- Global tilt angle (GTA): Angle between two lines connecting markers on the spinous processes of C7, L5 and the midpoint of markers on the greater trochanters (Fig. 2: markers CV7, LV5, R_FTC, L_FTC; Fig. 3g).
- Hip, knee, and ankle angles: For hip flexion and ankle dorsiflexion, mean minimal values from both legs were analyzed. For knee flexion, mean minimal values during the stance phase from both legs were used.

These parameters were chosen for their close correspondence to established radiographic spino-pelvic and sagittal balance parameters. Specifically, PA relates to sacral slope (SS), LLA to lumbar lordosis (LL), TKA to thoracic kyphosis (TK), C7-L5-STS to sagittal vertical axis (SVA), AM-HA-STS to center of the auditory meatus to hip axis (CAM-HA), STA to odontoid hip axis (OD-HA), and GTA to global tilt (GT) [3, 17, 18].

All parameters were calculated in three-dimensional space to capture spinal motion across sagittal, axial, and coronal planes. This was performed by projecting the respective markers onto a dynamic trunk-based plane (defined by the following markers: SXS, SJN, CV7, TV2, LV1, LV3, LV5, and MAI) before the calculations were performed. The spino-pelvic and sagittal balance parameters were calculated using MATLAB software (vR2020b), while the joint angles were calculated using Visual3D software (v6).

## Statistical analysis

Group demographic differences were assessed using independent t-tests and chi-square tests. Changes within groups were analyzed using linear mixed models with likelihood ratio tests. Group differences in pre-walk measurements were tested using ANCOVA with age as a covariate. Other between-group differences were assessed using linear mixed models adjusted for age, using Wald and likelihood ratio tests. To control for multiple comparisons, the Benjamini–Hochberg procedure was applied to p-values from ANCOVA, likelihood ratio tests, and Wald tests. K-means clustering classified participants by the pattern and magnitude of parameter changes from initial- to end-walk phases. The number of clusters was evaluated using elbow and silhouette methods, and cluster stability was assessed using bootstrap resampling with the adjusted Rand index. All analyses were performed using Microsoft Excel for Mac (v16.94), R (v4.4.2) and Julia (v1.12). Statistical significance was set at *p* < 0.05.

## Results

A total of 33 participants in the study group (8 males, 25 females) and 31 controls (9 males, 22 females) were included in the study. The demographic characteristics of the participants are presented in Table 1.

**Table 1:** Demographic and radiological characteristics of enrolled participants

	Study group (N = 33)	Control group (*N* = 31)	P-value (t-test, chi-square test)
Age (years)	71.3 ± 6.4	63.1 ± 7.2	< 0.001
Sex Male Female	8 (24.2%) 25 (75.8%)	9 (29.0%) 22 (71.0%)	0.880
Height (cm)	162.8 ± 9.2	166.4 ± 9.0	0.120
Weight (kg)	79.2 ± 17.5	77.7 ± 13.6	0.703
Body mass index (BMI) (kg/m^2^)	29.8 ± 6.2	27.9 ± 3.6	0.135
PI-LL (°)	20.0 ± 15.1	−4.2 ± 6.2	< 0.001
SVA (mm)	68.4 ± 52.1	2.2 ± 20.1	< 0.001

Analysis of gait assessment in the study group revealed marked changes in several parameters during the transition from standing to walking, with most returning to baseline levels post-walk. No statistically significant changes were observed during dynamic phases (initial-, mid-, end-walk). Detailed results are presented in Table 2.

**Table 2:** Values of the measured parameters for participants in the study group and evaluation of changes from static to dynamic conditions, as well as changes during dynamic measurements

Study group (*N* = 33)	Pre-walk (static)	Initial-walk (dynamic)	Mid-walk (dynamic)	End-walk (dynamic)	Post-walk (static)	Change from static to dynamic* (likelihood ratio test)	Changes during all dynamic measurements only† (likelihood ratio test)
PA (°)	9.0 ± 7.4	10.7 ± 7.9	10.6 ± 7.8	10.7 ± 8.1	8.9 ± 7.3	1.7 (− 0.8 to 4.0); p = 0.233	1.000
LLA (°)	8.5 ± 8.2	8.5 ± 6.8	8.2 ± 6.7	8.4 ± 6.9	6.7 ± 4.8	0.0 (− 1.0 to 1.8); p = 0.589	1.000
TKA (°)	25.6 ± 6.8	26.6 ± 6.5	26.5 ± 6.5	26.5 ± 6.8	25.8 ± 6.6	1.0 (0.7 to 2.1); p = 0.001	1.000
C7-L5-STS (mm)	69.1 ± 50.6	138.7 ± 51.9	140.7 ± 63.1	142.7 ± 65.4	77.4 ± 63.1	69.6 (55.3 to 84.2); p < 0.001	0.070
AM-HA-STS (mm)	73.3 ± 43.2	133.1 ± 58.6	134.5 ± 69.7	132.6 ± 70.4	70.7 ± 57.7	59.8 (46.7 to 77.8); p < 0.001	0.079
STA (°)	5.6 ± 3.6	10.6 ± 5.1	10.8 ± 6.1	10.6 ± 6.1	5.5 ± 4.8	5.0 (3.8 to 6.5); p < 0.001	1.000
GTA (°)	49.4 ± 12.0	57.7 ± 11.8	57.9 ± 12.3	58.4 ± 12.5	50.4 ± 14.2	8.3 (5.4 to 11.0); p < 0.001	0.306
Hip flexion (°)	10.3 ± 12.9	6.4 ± 13.0	7.6 ± 12.8	7.9 ± 13.0	11.3 ± 13.6		0.433
Knee flexion (°)	15.0 ± 10.1	18.7 ± 7.5	18.7 ± 6.9	18.4 ± 7.2	15.8 ± 10.0		0.158
Ankle dorsiflexion (°)	9.4 ± 4.2	-4.7 ± 3.9	-5.7 ± 4.4	-5.4 ± 4.0	9.5 ± 3.6		0.201

^*^ Mean difference (95% CI); p-value

^†^ P-value

**Fig. 4:**
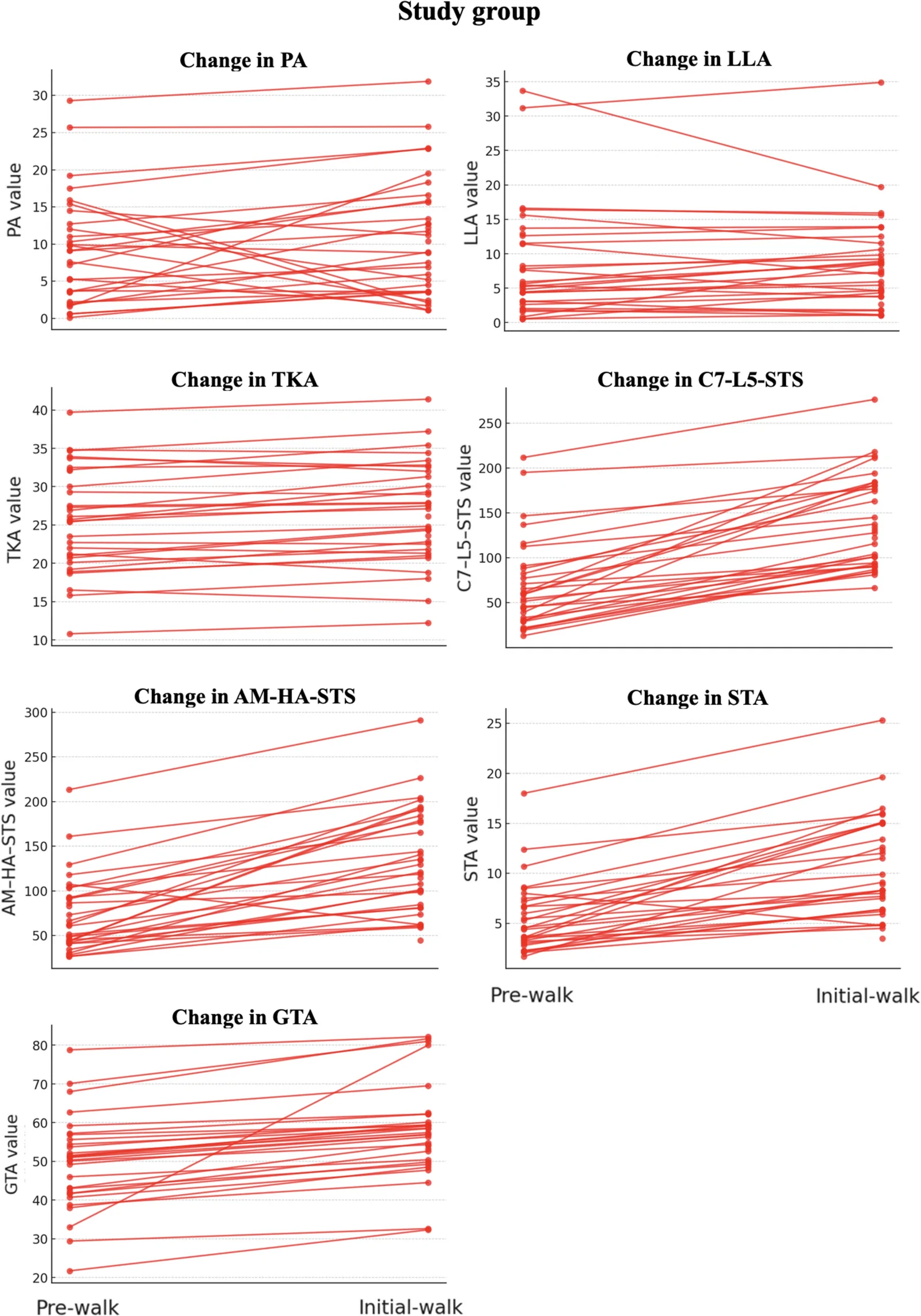
Parameter changes from pre-walk to initial-walk for each individual participant in the study group. Sagittal balance parameters (C7-L5-STS, AM-HA-STS, STA, GTA) increased consistently across most participants, while LLA and TKA remained largely unchanged and PA showed variable responses

Figure 4 illustrates individual parameter changes from pre-walk to initial-walk in the study group. Most parameters, particularly C7-L5-STS, AM-HA-STS, STA, and GTA, increased consistently across nearly all participants, with C7-L5-STS increasing over 100 mm in several individuals. In contrast, PA showed mixed responses, while LLA and TKA remained mostly unchanged.

**Fig. 5:**
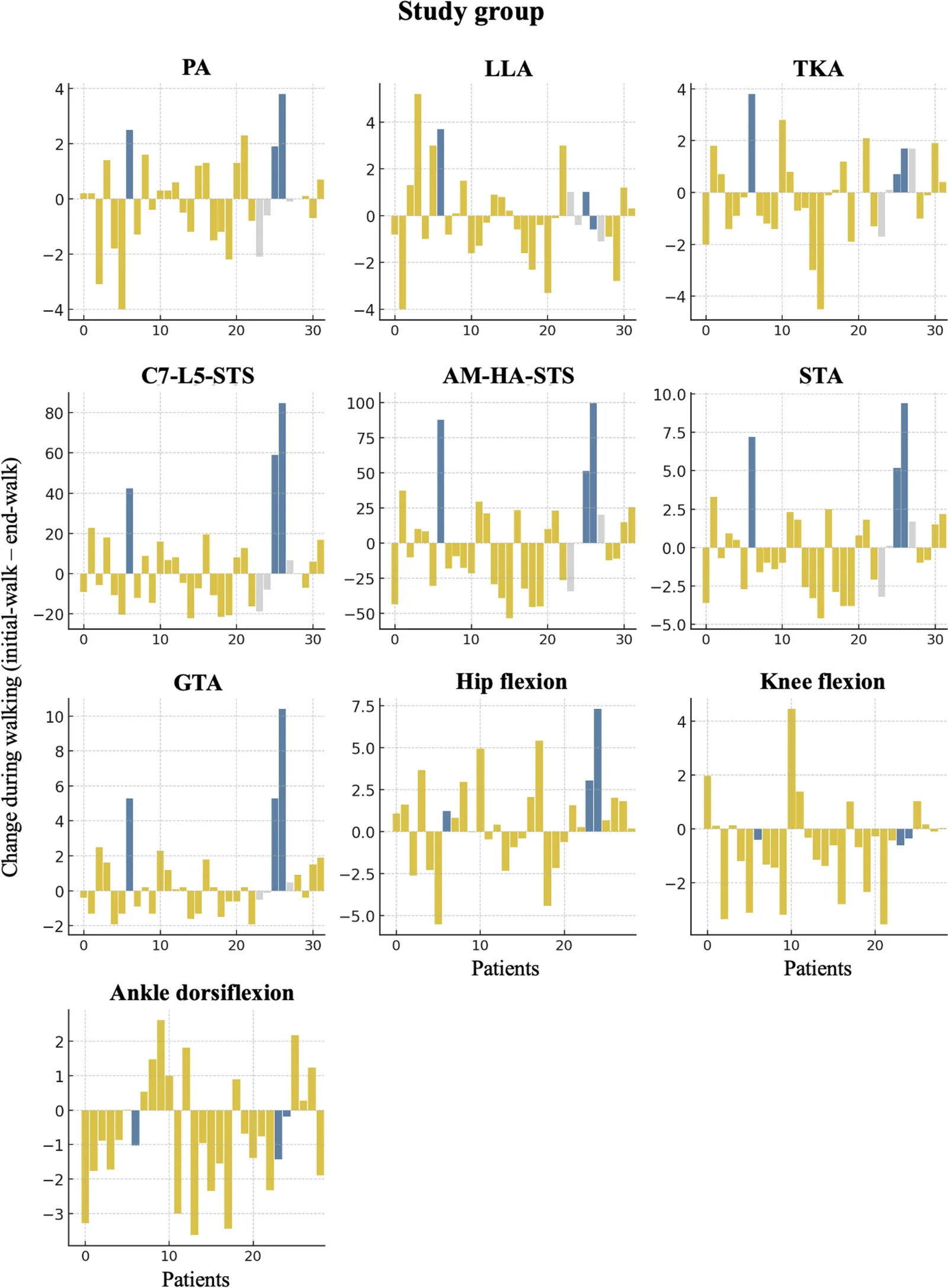
Parameter differences between the initial-walk and end-walk phases for each participant in the study group. Participants in the high-change group are shown in blue (*N* = 3), those with small changes in yellow (N = 26) and participants with incomplete data in gray (*N* = 4). In the high-change group, the largest changes occurred in sagittal balance parameters (C7-L5-STS, AM-HA-STS, STA, GTA), accompanied by consistent increases in PA, TKA, and hip flexion. The remaining participants demonstrated smaller and variable changes across all parameters

Figure 5 displays differences between the initial- and end-walk phases for each participant in the study group. Four participants were excluded from clustering analysis due to incomplete data. Participants with greater changes are marked in blue (N = 3), those with smaller changes in yellow and those with incomplete data in gray. The largest changes in the high-change group were seen in C7-L5-STS (up to + 84.7 mm), AM-HA-STS (up to + 99.7 mm), STA (up to + 9.4°), and GTA (up to + 10.4°). In contrast, remaining participants demonstrated smaller changes, frequently in the opposite direction. PA, LLA, TKA, and lower limb joint angles exhibited smaller, more uniform changes.

Controls similarly showed notable changes in several parameters during the transition from standing to walking, with most parameters returning to baseline levels after walking. During dynamic phases, only TKA showed statistically significant changes (*p* = 0.030). Detailed results are shown in Table 3.

**Table 3:** Values of the measured parameters for controls and evaluation of changes from static to dynamic conditions, as well as changes during dynamic measurements

Control group (*N* = 31)	Pre-walk (static)	Initial-walk (dynamic)	Mid-walk (dynamic)	End-walk (dynamic)	Post-walk (static)	Change from static to dynamic* (likelihood ratio test)	Changes during all dynamic measurements only† (likelihood ratio test)
PA (°)	6.0 ± 3.6	6.8 ± 4.2	7.1 ± 4.5	7.2 ± 4.0	6.0 ± 3.6	0.8 (0.0 to 1.6); p = 0.098	1.000
LLA (°)	12.2 ± 7.6	12.0 ± 6.0	11.4 ± 6.4	11.7 ± 6.4	11.8 ± 7.6	− 0.2 (− 1.5 to 1.2); p = 0.834	1.000
TKA (°)	27.0 ± 6.2	28.9 ± 5.3	28.4 ± 5.5	28.3 ± 5.5	26.3 ± 6.2	1.9 (1.3 to 3.1); p < 0.001	0.030
C7-L5-STS (mm)	20.9 ± 18.8	69.2 ± 19.7	70.3 ± 21.6	72.0 ± 22.6	22.7 ± 15.8	48.3 (40.5 to 56.2); p < 0.001	0.133
AM-HA-STS (mm)	53.0 ± 29.9	66.4 ± 34.8	66.2 ± 32.6	71.3 ± 33.4	57.1 ± 31.1	13.4 (− 2.6 to 29.2); p = 0.121	0.065
STA (°)	3.8 ± 2.1	4.8 ± 2.1	4.8 ± 2.0	5.2 ± 2.2	4.2 ± 2.2	1.0 (0.0 to 2.1); p = 0.084	1.000
GTA (°)	39.0 ± 8.8	47.0 ± 8.4	47.0 ± 9.1	47.0 ± 9.1	38.2 ± 9.2	8.0 (7.0 to 9.2); p < 0.001	1.000
Hip flexion (°)	5.1 ± 7.3	-2.3 ± 7.5	-2.3 ± 7.4	-2.7 ± 7.7	4.5 ± 7.0		1.000
Knee flexion (°)	8.2 ± 4.5	13.6 ± 4.2	13.4 ± 4.2	13.5 ± 3.9	8.5 ± 4.8		1.000
Ankle dorsiflexion (°)	7.7 ± 2.9	-8.4 ± 4.9	-8.9 ± 5.0	-9.2 ± 5.5	8.3 ± 3.2		0.405

^*^ Mean difference (95% CI); *p*-value

^†^ P-value

**Fig. 6:**
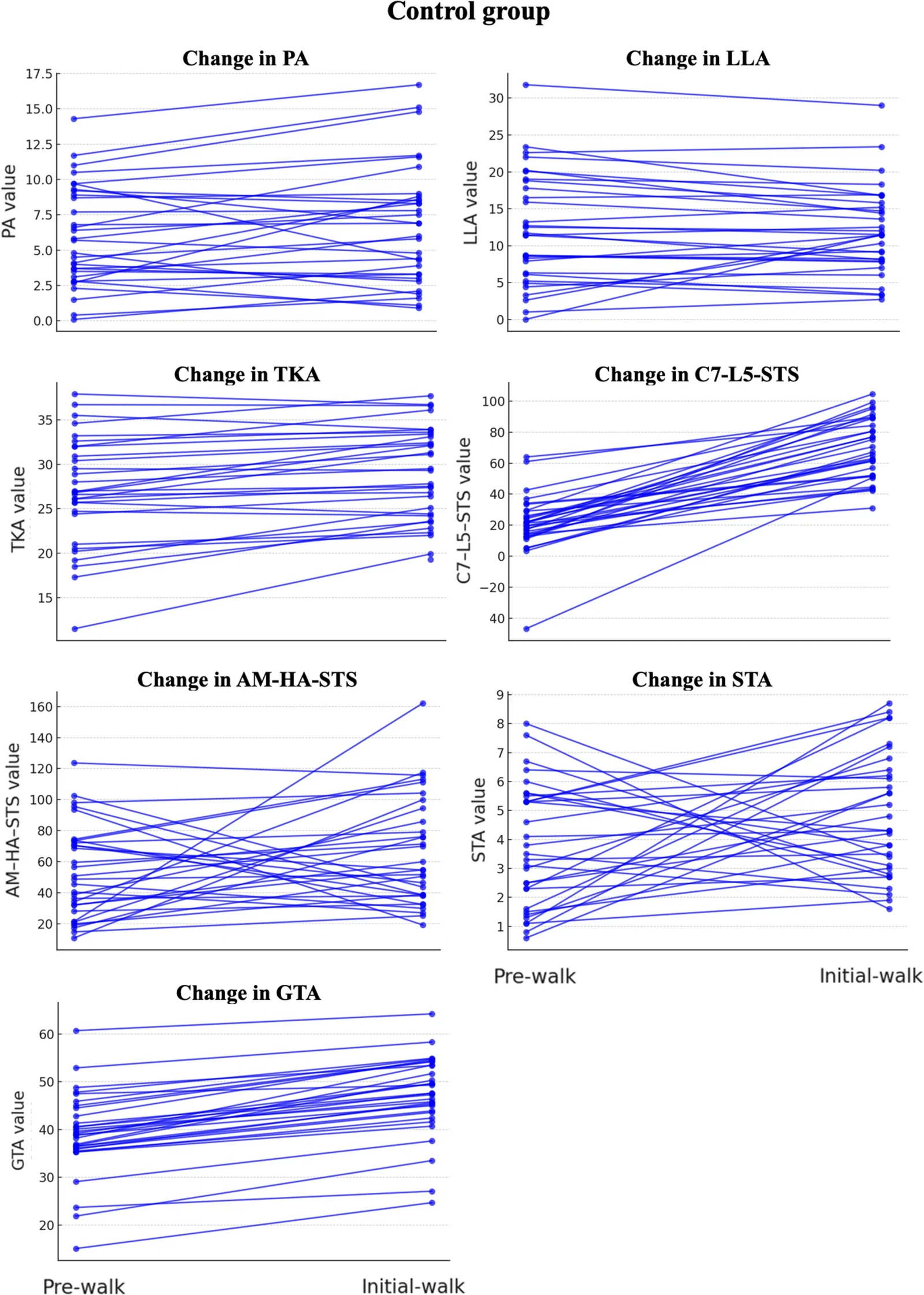
Parameter changes from pre-walk to initial-walk for each individual participant in the control group. C7-L5-STS and GTA generally increased across participants, TKA showed small but consistent increases, while PA, AM-HA-STS, and STA showed variable responses and LLA remained largely unchanged

Figure 6 presents individual parameter changes from pre-walk to initial-walk for all 31 controls. Similar to the study group, C7-L5-STS and GTA generally increased, with C7-L5-STS rising by more than 70 mm in some cases. PA, AM-HA-STS, and STA exhibited mixed responses, LLA remained stable and TKA showed small but consistent increases.

**Fig. 7:**
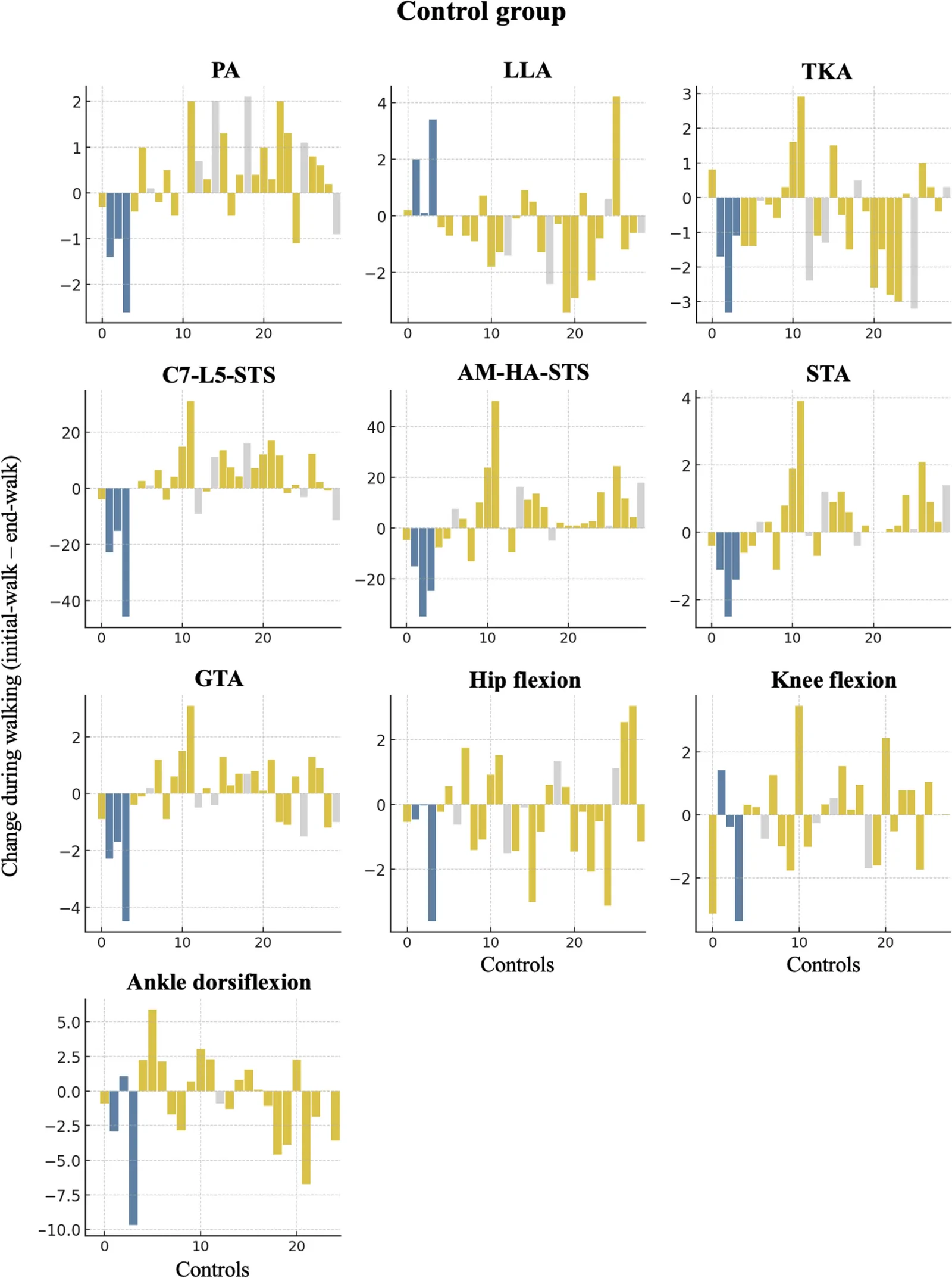
Parameter differences between the initial-walk and end-walk phases for each control participant. Participants in the high-change group are shown in blue (*N* = 3), those with small changes in yellow (*N* = 21), and participants with incomplete data in gray (*N* = 7). In the high-change group, the largest changes occurred in sagittal balance parameters (C7-L5-STS, AM-HA-STS, STA, GTA), showing decreases consistent with improved sagittal balance, accompanied by decreases in PA, LLA, TKA, and hip flexion. The remaining participants demonstrated slight increases in sagittal balance parameters and PA, with variable changes in other parameters

Figure 7 shows changes between initial- and end-walk phases for each control participant. Seven controls were excluded from clustering due to incomplete data. Participants with greater changes are marked in blue (N = 3), those with smaller changes in yellow and those with partial data in gray. Unlike the study group, the high-change controls were defined by decreases in measured parameters. Overall, changes across controls were highly variable: C7-L5-STS ranged from –45.6 mm to + 31.3 mm, AM-HA-STS from –34.9 mm to + 50.0 mm, STA from –2.5° to + 3.9° and GTA from –4.5° to + 3.1°.

Participants in the study group walked significantly shorter distances than the control group (17.2 ± 3.8 vs. 20.9 ± 3.5, respectively; *p* < 0.001). This difference remained statistically significant after adjusting for age using ANCOVA (F(1, 61) = 7.13, *p* = 0.010). Age-adjusted comparisons between participants in the study and control groups across different gait assessment phases are presented in Table 4.

**Table 4:** Differences between study and control groups, with p-values adjusted for age: for pre-walk (static) measurements; for changes from static to dynamic measurements; for all dynamic measurements throughout the entire walking period; and for additional changes observed from initial-walk to mid-walk and end-walk measurements

Comparison between study and control groups	Pre-walk (static) measurements* (ANCOVA)	Changes from static to dynamic* (likelihood ratio test)	All dynamic measurements* (Wald test)	Changes at mid-walk† (Wald test)	Changes at end-walk† (Wald test)
PA (°)	3.0 (− 0.2 to 6.2); p = 0.264	0.9 (− 1.5 to 3.3); p = 0.637	3.7 (0.1 to 7.3); p = 0.043	0.798	0.705
LLA (°)	− 3.7 (− 7.7 to 0.3); p = 0.258	0.2 (− 2.0 to 2.4); p = 0.637	− 3.3 (− 7.4 to 0.8); p = 0.109	0.830	0.725
TKA (°)	− 1.4 (− 4.5 to 1.7); p = 0.652	− 0.9 (− 2.2 to 0.4); p = 0.189	− 2.0 (− 6.5 to 2.5); p = 0.372	0.870	0.380
C7-L5-STS (mm)	48.2 (27.0 to 69.4); p = 0.005	21.3 (3.4 to 39.2); p = 0.020	70.2 (49.1 to 91.3); p < 0.001	0.974	0.781
AM-HA-STS (mm)	20.3 (0.0 to 40.6); p = 0.209	46.4 (21.0 to 71.8); p < 0.001	65.4 (28.0 to 102.8); p = 0.001	0.697	0.725
STA (°)	1.8 (0.3 to 3.3); p = 0.107	4.0 (2.3 to 5.7); p < 0.001	5.8 (3.1 to 8.5); p < 0.001	0.961	0.781
GTA (°)	10.4 (5.4 to 15.4); p = 0.006	0.3 (− 2.4 to 3.0); p = 0.944	11.0 (4.7 to 17.3); p = 0.001	0.984	0.380
Hip flexion (°)	5.2 (− 0.8 to 11.2); p = 0.209		9.7 (2.7 to 16.7); p = 0.007	0.865	0.380
Knee flexion (°)	6.8 (2.5 to 11.1); p = 0.077		5.1 (0.8 to 9.4); p = 0.022	0.959	0.438
Ankle dorsiflexion (°)	1.7 (− 0.3 to 3.7); p = 0.586		3.5 (− 0.8 to 7.8); p = 0.109	0.878	0.907

^*^ Mean difference (95% CI); *p*-value

^†^ P-value

## Discussion

Sagittal balance is a complex biomechanical concept requiring comprehensive evaluation. To obtain a detailed understanding of its dynamics during gait, we analyzed a complete gait cycle from standing to walking and back to standing. The groups were demographically similar except for age, which is not surprising, as advancing age is an important factor in the development of sagittal imbalance [19]. In addition, the presence of back pain in controls may have influenced gait dynamics and reduced the contrast between physiologic and pathologic gait patterns.

We found that the majority of sagittal balance changes occurred during the transition from standing to walking. In the study group, all sagittal balance parameters increased significantly: C7-L5-STS by an average of 69.6 mm, AM-HA-STS by 59.8 mm, STA by 5.0°, and GTA by 8.3° (all *p* < 0.001) (Table 2). Changes in spino-pelvic parameters were smaller, with PA increasing by 1.7° (*p* = 0.233) and TKA by 1.0° (*p* = 0.001). Individual participant data (Fig. 4) showed consistent increases in sagittal balance parameters, small but consistent increases in TKA, no changes in LLA and variable changes in PA. These findings suggest that the decrease of sagittal balance during gait initiation occurs in nearly all participants in this group, mainly due to increased thoracic kyphosis, with reduced pelvic retroversion contributing in some cases. Although a few participants exhibited increased pelvic retroversion, overall sagittal balance decreased across all cases. In the control group, changes in sagittal balance were less pronounced and reached statistical significance in only two parameters (Table 3). Among spino-pelvic parameters, only TKA increased significantly. Individual control participant data (Fig. 6) revealed patterns similar to the study group, except for AM-HA-STS and STA, which also incorporate head and neck positioning. This suggests mild sagittal balance decrease at gait initiation in the control group, partly compensated by increased cervical lordosis. These observations indicate that while some forward trunk inclination at gait initiation is physiological, its larger magnitude is clinically important and reflects impaired sagittal balance compensation. Comparing leg joint angles between standing and walking is not meaningful due to different biomechanical conditions.

During walking (initial- to end-walk), the study group exhibited only minor, non-significant changes in all parameters (Table 2). However, individual analysis (Fig. 5) identified three participants with greater overall changes, all of whom experienced significant decrease in sagittal balance. These participants showed a consistent reduction in pelvic retroversion, increased thoracic kyphosis, increased hip flexion, decreased knee flexion, and ankle dorsiflexion. Others showed variable changes, with some improvements observed across all parameters. These improvements may reflect enhanced blood flow, increased muscle temperature and improved neuromuscular activation during walking, potentially contributing to better muscle performance. The heterogeneity among individual participants likely explains the absence of statistically significant changes at the group level. Notably, two of the three participants with greater decrease had markedly elevated SVA and PI-LL values compared to the group average (SVA: 170.1 mm and 168.4 mm, with 68.4 mm average; PI-LL: 60.0° and 36.0°, with 20.0° average), suggesting that advanced degenerative spinal disease may contribute to greater sagittal balance decrease during walking. In the control group, changes during walking were minor across all parameters, with only TKA improving significantly (Table 3). Individual control data (Fig. 7) identified three participants who exhibited more pronounced changes, but unlike in the study group, these changes reflected improvements rather than decreases. These improvements were mainly due to increased pelvic retroversion, increased lumbar lordosis, and reduced thoracic kyphosis. Among the rest, changes were minor and less variable than in the study group, with slight increases in sagittal balance parameters and PA, and small decreases in LLA and TKA. Given the small size of the high-change subgroups, these observations should be considered exploratory and hypothesis-generating.

After walking, parameter values in both groups generally returned close to baseline (Table 2 and 3), indicating that compensatory mechanisms are more effective in a standing position than during walking.

The study group walked slower and covered significantly fewer lengths (17.2 ± 3.8) than controls (20.9 ± 3.5, *p* < 0.001). Although this difference could partly be explained by the higher average age in the study group, given that advancing age is associated with slower walking speed [20], the difference remained statistically significant after adjusting for age (*p* = 0.010). This finding suggests that sagittal imbalance and related spinal pathology may contribute to reduced gait speed, potentially through a combination of axial pain, altered gait kinematics [21], as well as increased muscular demands during sustained activation of compensatory mechanisms.

Significant group differences were observed in pre-walk (static) measurements after adjusting for age in C7-L5-STS and GTA (Table 4), with significantly more pathological sagittal balance parameters in the study group, consistent with inclusion criteria (elevated PI-LL and SVA). Although differences in spino-pelvic parameters and leg joint angles were not significant, average values tended to be more pathological among the study group. The absence of statistical significance in these parameters may be attributed to the substantial variability within the study group, evident from higher standard deviations. During the transition from standing to walking, the study group showed significantly greater decrease in sagittal balance parameters compared to controls, while changes in spino-pelvic parameters did not differ significantly between groups (Table 4). Therefore, the exact compensatory mechanisms responsible for this greater increase remain unclear. These findings further support the conclusion that the magnitude of sagittal balance decrease at gait initiation is more indicative than its presence, as some degree of decrease is physiological. Overall, this suggests that radiographic indicators of sagittal imbalance are associated with greater magnitude of sagittal balance decrease at the onset of walking. During walking (initial-, mid-, and end-walk) there were significant differences between groups in the absolute values of all sagittal balance parameters, PA, hip, and knee angles (Table 4). Although LLA was more pathological in the study group, the difference was not significant (Table 2 and 3). When analyzing the dynamics of changes during walking, specifically, changes from initial- to mid- and end-walk, no statistically significant differences were found between groups (all p > 0.05). These findings support the conclusion that significant decrease of sagittal balance during walking occurs only in a subset of patients and is therefore not detectable at the group level.

Severijns et al. similarly used motion capture system to study patients with sagittal imbalance and controls, though they used a shorter walking distance (10 m) [11]. They found increased forward trunk tilt and reduced pelvic retroversion at gait initiation, which was more pronounced in patients. Their findings are consistent with our results. Similar results were reported by Arima et al. and Rebeyrat et al., although their studies did not include control groups [10, 22]. Shiba et al. similarly observed a decrease in sagittal balance and reduced pelvic retroversion at gait initiation [7], however, unlike our study, they reported a further decrease during continuous walking on a treadmill until fatigue. This difference may be explained due to more severe deformity in their patients (mean SVA: 123 mm vs. our 68.4 mm), and longer walking duration. Miura et al. similarly described significant decrease in sagittal balance during walking to fatigue [12], again likely due to more severe deformity (their SVA: 142 mm) and longer walking duration. Kamata et al. also reported a significant increase in forward trunk tilt after just three minutes of walking, primarily attributed to decreased pelvic retroversion [9]. We did not observe similar changes at a group level, again likely due to the less severe spinal deformity (their SVA: 193 mm). To our knowledge, no previous study analyzed changes in spino-pelvic and sagittal balance parameters during the transition from walking back to standing. By including this measurement, our study demonstrates that compensatory mechanisms are more effective in a standing position, restoring sagittal balance to near baseline values after walking.

The major findings of this study are that compensatory mechanisms active during standing decline significantly as soon as individuals transition into walking, primarily due to increased thoracic kyphosis and, in some participants, decreased pelvic retroversion. While these changes were observed in both groups, sagittal balance decrease was more pronounced in the study group. During walking, most parameters did not exhibit statistically significant changes, except for TKA in the control group. These findings suggest that meaningful decrease in sagittal balance during walking occurs predominantly in a smaller subgroup of participants within the study group as well as control group, which may not be clearly reflected at the group level (Figs. 5 and 7). After walking, compensatory mechanisms again restored sagittal balance, albeit somewhat less effectively, possibly due to muscle fatigue.

Limitations of this study include several methodological considerations that could be addressed in future research. Using more homogeneous groups, particularly regarding age, would reduce potential age-related effects, while a more balanced gender distribution could improve generalizability. Including a control group without back pain would allow more valid comparisons. During walking, participants followed an elliptical path with turns in the same direction, which may have caused slight lateral trunk tilt. While this is a standard approach, some authors have proposed using a figure-eight pattern to alternate turning directions [23]. Furthermore, the walking path was limited to 7 m due to camera placement constraints, which is shorter than the recommended 10 to 30 m. Extending walking beyond six minutes could reveal a further decrease in sagittal balance. No direct or indirect measures of muscle fatigue were collected; therefore, any fatigue-related interpretations remain speculative. Finally, analyzing more than three steps per phase would improve representativeness of the results but was sometimes restricted by marker signal dropout.

## Conclusion

Sagittal balance decreases significantly at gait initiation, with a larger decline in patients with degenerative spine disease than in controls, and remains relatively stable during continuous walking in both groups. These findings indicate that compensatory mechanisms are more efficient in a standing position than during walking. Further research is needed to identify prognostic factors associated with more pronounced sagittal balance decrease during continuous walking, as such changes appear to affect only a subgroup of patients.

## Data Availability

The data that support the findings of this study are available from the corresponding author upon reasonable request.
